# Use of mental techniques for competition and recovery in professional athletes

**DOI:** 10.1007/s00508-016-0969-x

**Published:** 2016-03-01

**Authors:** Mohammad Keilani, Timothy Hasenöhrl, Immanuel Gartner, Christoph Krall, Johannes Fürnhammer, Fadime Cenik, Richard Crevenna

**Affiliations:** 1 Department of Physical Medicine and Rehabilitation, Medical University of Vienna, Währinger Gürtel 18–20, 1090 Vienna, Austria; 2 Section of Medical Statistics, Core Unit for Medical Statistics and Informatics, Medical University of Vienna, Vienna, Austria

**Keywords:** Professional, Athletes, Team sports, Mental techniques, Austrian

## Abstract

**Background:**

The present study aims to describe knowledge about and usage of mental techniques to prepare before competitions and after sport-associated injuries (SAIs) by professional athletes (team sports) in Austria.

**Methods:**

In this cross-sectional study, 191 professional athletes (basketball, football, hockey, ice hockey, and volleyball teams, m:f = 142:49, 24 ± 5, 18–39 years) filled in a questionnaire assessing socio-demographic data, duration/frequency of sport practice, rate and severity of SAIs. Furthermore, the use of mental techniques and of spiritual practices before competitions and for recovery after SAI was assessed. The use of mental techniques before competitions and after SAI was correlated with socio-demographic data, duration and frequency of sport practice, and injury patterns of SAIs of the last 24 months.

**Results:**

Approximately, 96 % reported knowledge about at least one mental technique. Only 13 participants used them for regeneration after SAI. Approximately, 31 % of males and 13 % of females reported the use before competitions (*p* = 0.017). 54 % of participants using spiritual practices used mental techniques before competitions, whereas only 13 % of participants not using spiritual practices used them (*p* < 0.001). 67 % of participants not using mental techniques before competitions and 88 % using them believed in the effectiveness of mental techniques in the regeneration after a SAI (*p* = 0.03). A significant increase of the probability of using mental techniques before competition with increasing age was found [Odds ratio (OR) = 1.101, confidence interval (CI) = (1.03, 1.18), *p* = 0.006].

**Conclusion:**

Mental techniques seem to be well-accepted but rarely used among professional athletes. Further studies are needed to give new information about this relevant topic in professional sports.

## Introduction

Different professional sports are often associated with different behavioral, physical, and psychological symptoms [1]. Examples of such symptoms are sleep disorders, headache, increased muscle tension, negative thoughts, and difficulties concentrating [1]. Psychological issues, like distress, are known to be able to deteriorate performance of athletes during competition [1]. Additionally, athletes are exposed to stress from performance demands imposed by media, their teams, fans, and themselves [2]. Furthermore, distress is supposed to be an important antecedent to sport-associated injuries (SAIs) [3–5]. SAIs are often accompanied by substantial psychological effects (distress, anxiety) [5–7].

Different mental techniques might counteract distress of athletes in enhancing their mental skills and therefore their ability to cope with stressors. Mental techniques (such as hypnosis [8–10], progressive muscle relaxation [11–13], cognitive interventions [11], biofeedback [14–16], autogenic training [17, 18], breathing techniques [19], imagery/visualization [12], and others) have been shown to be effective to improve performance in sports, and to facilitate mental recovery from SAI. There are several studies that examined mental skills of athletes associated with success in the Olympic and national/international championships. Nevertheless, most of studies were based on self-reports of athletes and associated with single competitions like Olympic Games and international championships [20]. To our knowledge, no studies have explored the use of mental techniques by professional team athletes before competition and for recovery from SAIs in Middle Europe, yet. Furthermore, there seems to be a need for studies to determine if team athletes really use well-accepted mental techniques like hypnosis progressive muscle relaxation [11–13], cognitive interventions [11], biofeedback [14–16], autogenic training [17, 18], breathing techniques [19], imagery/visualization [12]. Therefore, the purpose of the present study was to explore the use of mental techniques by professional athletes, all of them were team players (of Austrian basketball, football, hockey, ice hockey, and volleyball teams), and to assess the use of mental techniques depending on socio-demographic characteristics, sport experience, and SAIs.

## Methods

The present explorative cross-sectional study took place in cooperation with several Austrian 1st division teams (basketball, football, hockey, ice hockey, and volleyball). A total of 200 professional athletes of the included teams were asked personally to participate. All of them accepted to participate in this study, and were active professional athletes (the duration of their professional experience was longer than 24 months). Exclusion criteria were systemic diseases, pregnancy, participation in other studies, and age below 18. In all, nine subjects answering this invitation were excluded from the study (3 were younger than 18, 6 were included in other studies). Therefore, 191 (*n* = 191) athletes were included.

The present study was approved by the ethics committee of the Medical University of Vienna (EK-Nr. 1890/2012). After explanation of the aim of the study and guarantee of confidentiality, all participants received an anonymous self-administered questionnaire to fill in. The following socio-demographic data of participants were assessed: age, size, weight, partner status, and usual residence (urban/rural). To evaluate sports attitudes, the participants were asked for duration of sport practice (years of active professional practice, competitions/week, weekly training time (hours/week)). Furthermore, the participants were asked for the frequency of musculoskeletal and superficial injuries (SAIs) during the last 24 months. The kind of the most serious injury was reported and grouped into three severity degrees (I mild: laceration; II moderate: contusion, strain/sprain, bruise; III severe: fracture, ligament rupture). Socio-demographic data, sport behavior, injury patterns of SAIs were correlated with the knowledge about (Which mental technique have you heard of?) and the use of different mental techniques (hypnosis, breathing techniques, autogenic training, imagery/visualization, tai chi/qi gong, progressive muscle relaxation, biofeedback, and others) for preparation before competitions and for recovery after SAIs were assessed for correlation with other reported data.

### Statistical methods

Demographic data and discipline of respondents are reported by means and standard deviations respectively frequencies. Odds ratios for usage of mental techniques depending on the covariates (age, education, sports experience (years), total training time (hours/week), number of trainings, and number of competitions) were modeled by generalized linear models with logistic regression. Correlation between usage of mental techniques and qualitative variables was analyzed with chi-squared tests (sex, partner status, residency, and other spiritual practices) or Fisher’s exact test (kind of sport). Due to the explorative character of the study no adaptation of the *p*-value was performed [21]. All calculations were performed with R 3.02 and R Studio.

## Results

Demographic data and sports of respondents are presented (Table 1).

**Table 1:** Demographic data and types of sports of the study population (*n* = 191 consecutive professional athletes, m:f = 142:49, age: mean = 24 ± 5a, min = 18, max = 39, body mass index: mean = 25 ± 4 kg/m^2^ min = 18, max = 56)

Characteristic	Number of patients	% of patients
*Sex*		
Male	142	74
Female	49	26
*Residency*		
Urban	147	77
Rural	23	12
Refused to answer this question	21	11
*Partnership*		
Relationship	84	44
Singles	72	38
Refused to answer this question	20	10
Married	15	8
*Sports*		
Basketball	54	30
Football	50	27
Volleyball	45	25
Ice Hockey	18	10
Field Hockey	15	8

Table 2 gives the absolute and relative frequencies of participants knowing each included mental technique (knowledge). Approximately, 96 % of participants reported their knowledge about at least one mental technique.

**Table 2:** Absolute (*n*) and relative (percentage) frequencies of participants (*N* = 191) with knowledge about each of the included mental techniques

Mental technique	Unknown *n* (percentage of all answers)	Known *n* (percentage of all answers)	Number answer *n* (percentage of everyone asked; *N* = 191)
Autogenic training	62 (39 %)	99 (61 %)	30 (16 %)
Breathing techniques	47 (29 %)	113 (71 %)	31 (16 %)
PMR	104 (65 %)	57 (35 %)	30 (16 %)
Hypnosis	56 (35 %)	105 (65 %)	30 (16 %)
Tai chi/qi gong	72 (45 %)	89 (55 %)	30 (16 %)
Imagery/visualization	71 (44 %)	90 (56 %)	30 (16 %)
Others	157 (98 %)	4 (2 %)	30 (16 %)
Biofeedback	118 (62 %)	45 (24 %)	28 (15 %)
Any of these	7 (4 %)	154 (96 %)	30 (16 %)

*PMR* progressive muscle relaxation

Only 13 participants reported to have used mental techniques for regeneration after an injury/SAI. Therefore, we focused on the use of mental techniques in preparation before competitions; henceforth “use of mental techniques” will refer exclusively to usage in this respect.

Usage of mental techniques is presented in table 3. A total of 17 participants refused to answer this question.

**Table 3:** Usage of mental techniques for preparation before competitions

Characteristic	No	Yes
*Sex*		
Male	86	40
Female	42	6
*Residence*		
Urban	105	42
Rural	19	4
*Partnership*		
Relationship	63	21
Single	53	19
Married	9	6
*Sports*		
Basketball	39	13
Football	33	14
Volleyball	33	8
Ice Hockey	10	5
Field Hockey	8	4
*Spirituality*		
Yes	21	25
No	99	16

A significant increase of the probability of using mental techniques with increasing age was found [odds ratio (OR) = 1.101, confidence interval (CI) = (1.03, 1.18), *p* = 0.006]. Significantly more males than females reported to use mental techniques before competitions [OR = 3.25, 95 % CI = (1.36, 9.09), *p* = 0.017, Table 3].

Further sociodemographic factors [partner status: *p* = 0.48, residency: *p* = 0.39, education: OR = 1.042, 95 % CI = (0.76, 1.42), *p* = 0.792] showed no significant associations with the use of mental techniques.

Significantly more users than none users of other spiritual practices used mental techniques [*p* < 0.001, OR = 0.14, 95 % CI = (0.06, 0.32), table 3].

In comparison to inexperienced athletes, a significantly higher percentage of the athletes experienced in using mental techniques in preparation for competitions believed in the effectiveness of mental techniques in the regeneration after injuries [88 % vs. 67 %, OR = 0.3, 95 % CI = (0.10,0.78), *p* = 0.02].

Variables influencing the usage of mental techniques in preparation for competitions like sex, residence, partnership status, kind of sport and affinity to spirituality are presented in Table 2. The variable “sport” had no significant influence on the tendency to use mental techniques (*p* = 0.81). Furthermore, no significant correlation between the probability of using mental techniques and the factors sports experience (years), total training time (hours/week), number of trainings, and number of competitions per week was found (OR = 0.97–1.16, *p* = 0.09–0.97).

Participants were classified into those suffering injuries and those who did not. Mental techniques were used by 30 % of all participants with at least one injury and by 16 % of all participants with no injury. The difference was not significant [OR = 2.21, 95 % CI = (0.91, 6.26), *p* = 0.14]. Fractures and ruptures were counted as severe injuries, and a new variable assuming value 1 for participants with at least one severe injury in the past and 0 otherwise was defined. Out of all participants without a severe injury, 23 % used mental techniques, whereas 32 % of all patients with at least one severe injury used mental techniques. This difference was not found to be significant [OR = 1.49, 95 % CI = (0.74, 2.99), *p* = 0.34].

## Discussion

Mental techniques are used in sport primarily to enhance recovery from training and competition, to manage anxiety, and to improve performance [22, 23]. They have been shown to be effective in increasing concentration, enhancing motor skills, and improving ability to handle arousal and stress [22, 23]. Furthermore, a consensus statement from six different sports medicine associations indicated that psychological issues of athletes should be considered when treating and coordinating care of injured athletes [1].

The use of mental techniques seems to be widely accepted and well-described within professional athletes at times of big competitions like world championships or the Olympic Games [20]. For example, Gould et al. [20] performed a review on articles concerning psychological preparation of athletes for the Olympic Games. The authors concluded that factors associated with success seem to be certain psychological characteristics, cognitive and behavioral strategies, and personal dispositions. These factors include coping strategies (e.g., thought control, motion control, task focus, relaxed breathing, rational thinking, positive orientation, body awareness, confidence, ability to focus attention and control performance imagery) [20]. Furthermore, use of mental training seems to have a positive influence on success [20]. In comparison, the present study aimed to describe the usage of mental techniques by athletes during regular season training.

The imbalance of the study population is remarkable (142 men and 49 women). The imbalance of sex distribution in the present study results from the sport types investigated, because females are still underrepresented in certain professional sports (especially American football, Ice hockey, Basketball) in Austria.

In the present study, 96 % of participants reported that they knew (they have the knowledge about) at least one mental technique (Table 2).

Approximately, 67 % of participants not using mental techniques in preparation before competitions and 88 % using them before competitions reported to believe in the effectiveness of mental techniques in the regeneration after SAI. Nevertheless, only a minority of the study population reported the use of mental techniques for either recovery after SAI (7 %) or for preparation for competitions (25 %). A possible reason for these contradictory results might be that these professional athletes seem to have insufficient access to sports psychology and coaching in mental techniques in their individual teams. This lack of access to mental techniques might be due to limited awareness for psychological issues of the athletes themselves or of their team coaches.

In comparison, Mann et al. [2] evaluated the availability of sports psychologists and other mental health resources for athletes. The authors reported that the majority of athletes were rarely or never referred to sport psychologists for injury-related and non-injury-related problems [2]. In contrast, Gould et al. [20] explicitly described the implementation of different mental techniques in the preparation process for the Olympic Games but had to admit that their emphasis was mainly directed towards successful athletes.

The use of prayer and other religious-spiritual practices correlated significantly with the use of competition mental techniques before competitions. Some targets of spiritual techniques usually address stress reduction, relaxation, prevention and treatment of anxiety [23]. This indicated that spirituality might promote self-awareness of participants. Therefore, spiritual athletes might be more open to the use of mental techniques.

In the present study, increasing age significantly increased the probability of utilization of mental techniques. In contrast, sports behavior (duration of sport practice, years of active professional practice, competitions/week, and weekly training time) and type of sport were not associated with the use of mental techniques. This indicates that the use of mental techniques in older athletes might be associated with other factors than sport experience. In other studies, more experienced athletes were shown to have better mental skills compared with less experienced controls [20, 24]. Therefore, it remains controversial which factors might influence the probability of utilization of mental techniques in athletes [20, 24].

Male athletes reported to use mental techniques more often than female athletes (Table 3) and partnership status, education and residence did not correlate with use of mental techniques. The reasons for this were not investigated in the present study, which can be seen as a limitation. Further limitations were that only athletes in certain sport types were assessed, and therefore, conclusions on other populations cannot be drawn from the results of the present observation. Furthermore, availability and accessibility of possible mental interventions and techniques were not assessed in detail.

## Conclusion

Although psychological issues of athletes should be considered when treating and coordinating care of injured athletes [22], the current study revealed that mental techniques were well-accepted but rarely used from the study population, namely professional athletes (Austrian and International players) from Austrian 1st division teams.

Due to the fact that different mental techniques are known to counteract distress of athletes, to be effective to improve performance, and to facilitate mental recovery from SAI, their use should be of high interest to increase performance and results especially for professional teams. Therefore, further studies are urgently needed and should be designed with respect to the limitations of the present study to give new information about this—to our opinion—in the future quite relevant topic to optimize performance in professional sports.
